# Percutaneous radiofrequency ablation in the treatment of pulmonary malignancies: efficacy, safety and predictive factors

**DOI:** 10.18632/oncotarget.24270

**Published:** 2018-01-18

**Authors:** Tina Streitparth, Denis Schumacher, Robert Damm, Bjoern Friebe, Konrad Mohnike, Ortrud Kosiek, Maciej Pech, Jens Ricke, Florian Streitparth

**Affiliations:** ^1^ Department of Radiology, University Hospital Munich, Munich, Germany; ^2^ Department of Neurology, Clinic of Magdeburg, Magdeburg, Germany; ^3^ Department of Radiology, Otto-von-Guericke University Clinic Magdeburg, Magdeburg, Germany; ^4^ DTZ, Berlin, Germany

**Keywords:** lung malignancies, radiofrequency ablation, colorectal cancer, microwave ablation

## Abstract

**Purpose:**

The purpose of this study was to evaluate the efficacy, safety and predictive factors of RFA of primary and secondary lung malignancies.

**Patients and Methods:**

79 patients with 129 primary and secondary lung malignancies were enrolled in a retrospective study. We treated 74 pulmonary metastases of colorectal cancer, 13 malignant melanoma lesions, 13 renal cancer metastases, 5 primary lung malignancies and 24 tumors of other different entities. All patients were considered to be unsuitable candidates for surgery, radiotherapy or chemotherapy. The primary endpoint was local tumor control, secondary endpoints were overall survival, safety and predictive factors, e.g. distance to pleura, vessels and bronchi.

**Results:**

The median tumor size was 1.2 cm (0.5–3.0 cm). After a median follow-up of 14 months (3–81 months), the LTC was 85.3 %. There were 34 lesions (26.4%) with complete remission, 48 (37.2 %) partial remission, 28 (21.7%) stable disease and 19 lesions (14.7%) with progressive disease. We evaluated an OS of 27 months. Pneumothorax in 19 cases (14.7%) and pleural effusion in 2 cases (1.6 %) were the leading complications (CTCAE, 5 grade III adverse events). The only significant influence regarding the outcome after RFA was the initial tumor size (*p* = 0.01). Distance to vessel, bronchi, and pleura showed no significant effect (*p* = 0.81; *p* = 0.82; *p* = 0.80).

## INTRODUCTION

Surgical resection is the standard of care for early stage NSCLC, with overall 5-year survival rates ranging from 40% to 67% for stage I [1, 2] and from 25% to 55% for stage II [1]. Pulmonary metastasectomy is also the treatment of choice for lung metastases. However, the role of surgical resection is determined by the location, the number of the pulmonary lesions, different co-morbidities and advanced disease of the patients [3].

For high-surgical-risk patients, several studies reported promising results for minimally invasive therapies such as image-guided radiofrequency ablation (RFA) and laser-induced thermo-ablation (LITT) [4–7]. In the last years, newer local-ablative treatments like microwave ablation (MWA) and high-dose-rate (HDR) brachytherapy gained success in the treatment of pulmonary lesions. Considering organ-specific and tissue-related conditions in the lung that affect technical success, percutaneous tumor ablation is assured to be a feasible, safe and effective minimally invasive procedure [5, 8, 9].

Numerous studies have analyzed the outcome of RFA in the treatment of lung malignancies and have shown promising results regarding efficacy and safety. The aim of this single-center university study was, besides the assessment of efficacy and safety, the evaluation of the influence of possible predictive factors on outcome and safety.

## RESULTS

### Treatment characteristics and technical success

We treated 79 patients with 129 lung tumors in 117 sessions. The median diameter of all 129 lung tumors was 1.2 cm (0.5 to 3.0 cm; mean 1.3 cm). 41 lesions ranged from 0.5–1 cm in tumor size, 74 lesions ranged from 1– 2cm and 14 lesions ranged from 2–3 cm. Regarding the distance to a pulmonary vessel, 14 tumor lesions were directly adjacent (0–0.5 cm), 82 lesions showed a distance between 0.5 to 2 cm and 33 appeared > 2 cm. Regarding the distance to a bronchus, 8 lesions were 0–0.5 cm in distance, 61 were located 0.5–2 cm from the bronchus and 60 showed relevant distance (> 2 cm).

47 malignancies (36.4%) were spread in the upper lobe, 12 tumors (9.3%) were located in the intermediate lobe and 70 targets (54.3%) were part of the lower lobe in both lungs. Relating to the neighboring pleura, 10 lesions were directly adjacent to the pleura (0–0.5 cm) and 119 showed a distance > 1 cm.

Treatment was successfully completed in all 117 ablation procedures (100%) without any premature interruption. The median time of the complete procedure was 17 minutes (range, 10–30 minutes).

### Local tumor control, overall survival and predictive factors

**Figure 1:**
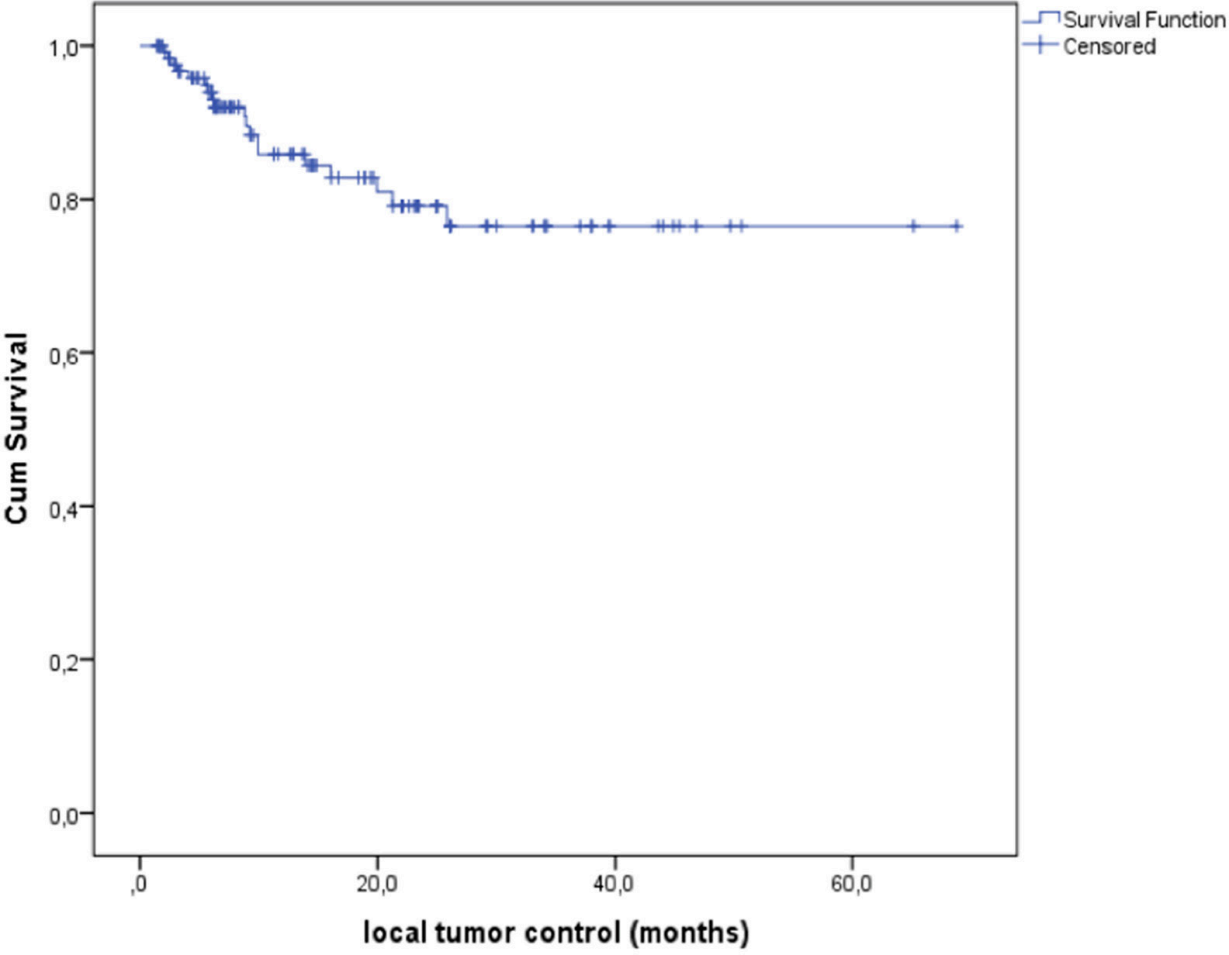
Local tumor control (LTC) was 85.3% after percutaneous CT-guided radiofrequency ablation (RFA) of primary and secondary pulmonary malignancies

There was a median follow-up period of 14 months (3–81 months) to examine LTC as the primary endpoint of this study. LTC was achieved in 110 target tumors (85.3%) (Figure 1). Regarding to RECIST 1.1, 34 lesions (26.4%) showed complete remission (CR), 48 tumors (37.2%) showed partial remission (PR), 28 cases (21.7%) were rated as stable disease (SD) and 19 (14,7%) treated lesions which resulted in progressive disease (PD) (Table 1).

**Table 1:** Median overall survival, local recurrence, local tumor control and the influence of possible predictive factors

**Total number of patients (*n*)**	**79**
**Number of lesions (*n*)**	**129**
**Median follow up (mo.)**	**14**
**Median overall survival (OS, mo.)**	**27**
**Median OS regarding tumor size:**	
**0.5–1 cm**	**26.2**
**1–2 cm**	**26.0**
**2–3 cm**	**16.3**
**Local tumor control (*n*; %)**	**110; 85.3**
**complete remission (CR)**	**34; 26.4**
**partial remission (PR)**	**48; 37.2**
**stable disease (SD)**	**28; 21.7**
**progressive disease (PD)**	**19; 14.7**
**Local recurrence (*n*; %) regarding *Tumor size***	***p* = 0.013**
**0.5–1 cm**	**7.3**
**1–2 cm**	**16.2**
**2–3 cm**	**28.6**
***Distance to pleura***	***p* = 0.807**
**< 1cm**	**2**
**≥ 1cm**	**17**
***Distance to vessel***	***p* = 0.812**
**0–5 mm**	**2**
**5–20 mm**	**12**
**> 20 mm**	**5**
***Distance to bronchi***	***p* = 0.822**
**0–5 mm**	**2**
**5–20 mm**	**10**
**> 20 mm**	**7**
**Subanalyze of colorectal cancer metastases:**	
**Median overall survival (OS)**	**13**
**Median time to recurrence (TTR)**	**8**

Subanalyze of colorectal cancer metastases

**Figure 2:**
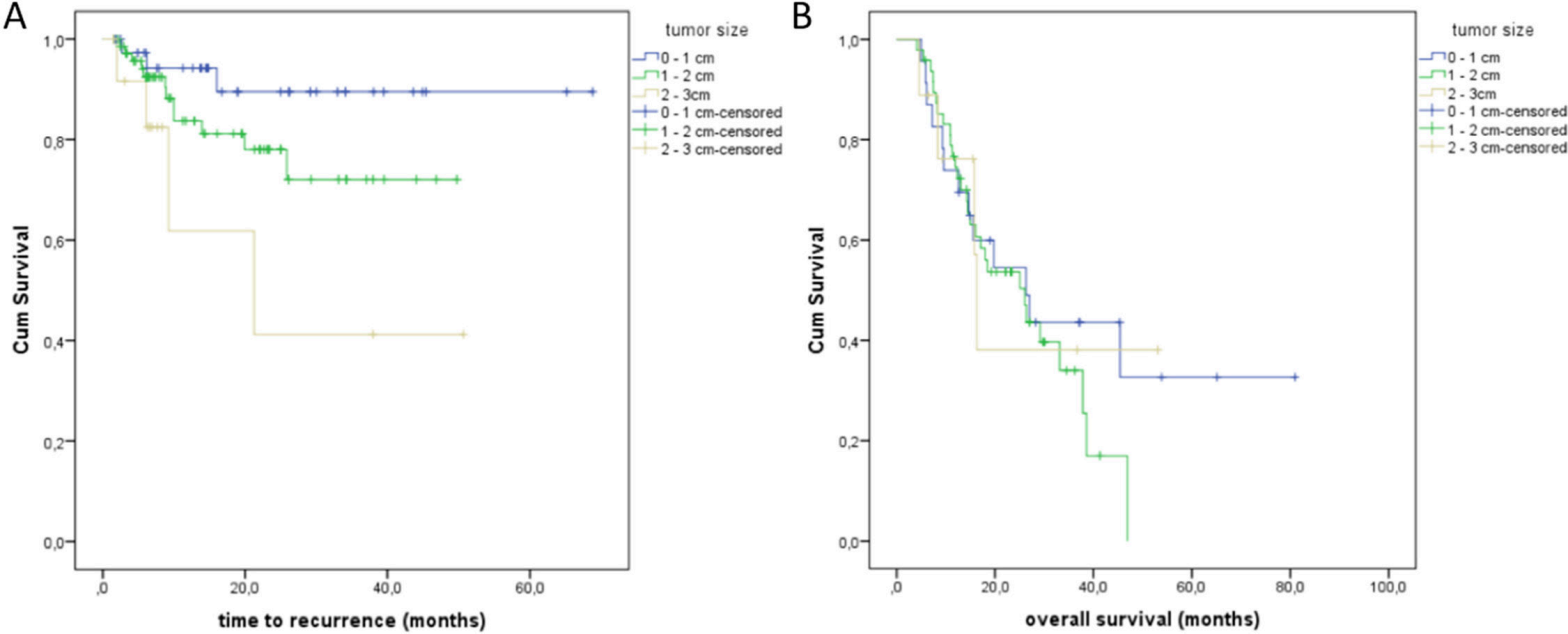
(**A)** Time to recurrence regarding the tumor size of the treated lung malignancies. (**B)** Overall survival (OS) depending on the tumor size; patients with tumor size of 0.5–1 cm showed a median OS of 26.2 months; patients with a tumor size of 1–2 cm and a median OS of 26.0 months; patients with a tumor size of 2–3 cm and a median OS of 16.3 months.

Validation of possible predictive factors stratifying the survival curves indicated that LTC was decreased in cases of larger initial tumor size. Lung malignancies between 2 and 3 cm in longest diameter presented local progression in 28.6%, 1 to 2 cm tumors in 16.2% and lesions ranging from 0.5 to 1 cm in only 7.3% (Figure 2A, Table 1). Size class demonstrated a significant influence (*p* = 0.013, log-rank; *p* = 0.029, Breslow) on local tumor progression. Overall survival was not significantly affected by initial target lesion size (*p* = 0.659, log-rank). Patients with an initial tumor size from 0.5 to 1 cm showed a median OS of 26.2 months; in 1 to 2 cm and 2 to 3 cm tumor sizes a median OS of 26.0 and 16,3 months was found respectively (Figure 2B, Table 1). Cox regressions confirmed initial target lesion size as an important impact factor (*p* = 0.012).

**Figure 3:**
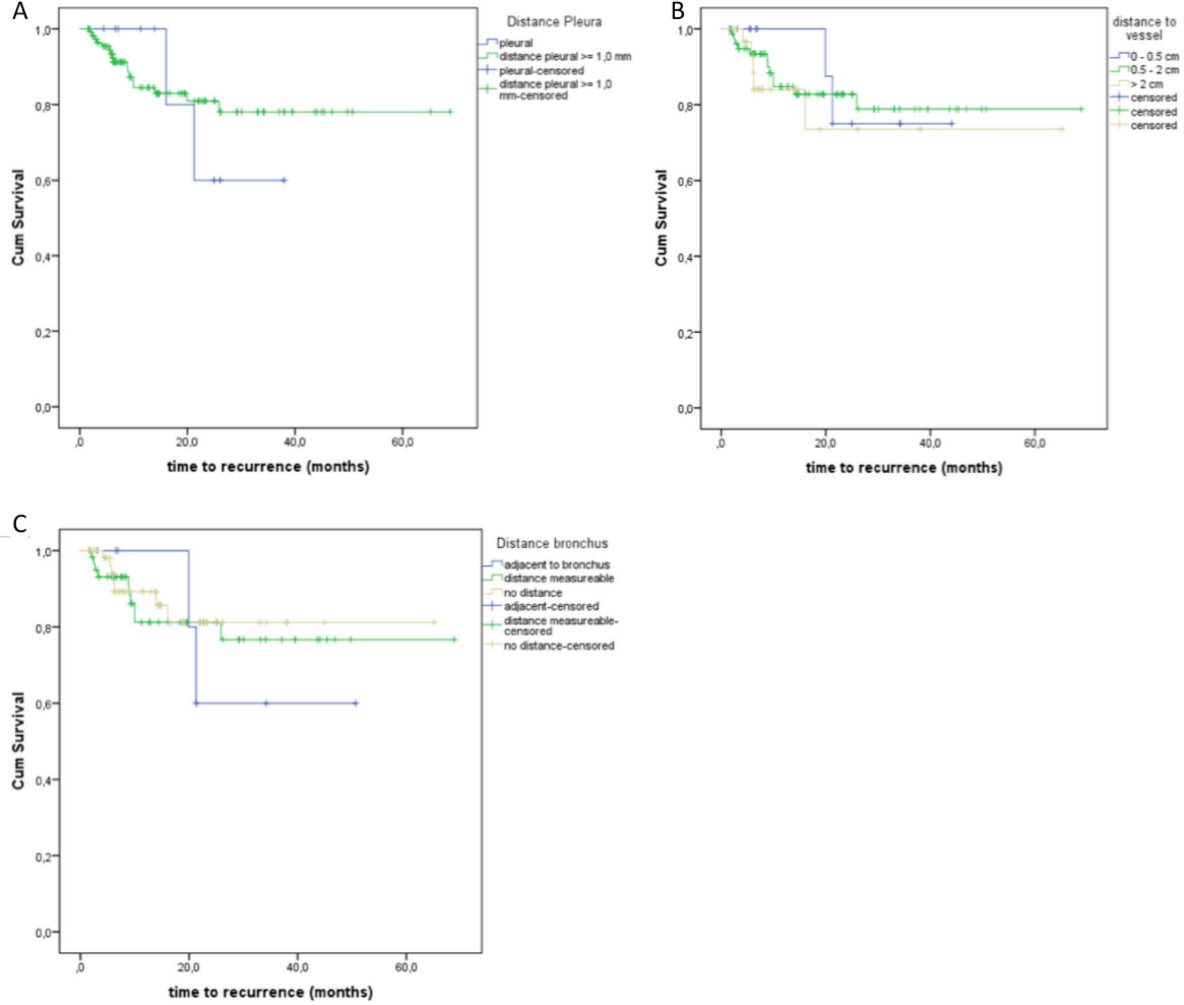
(**A)** Local recurrence regarding the influence of the distance to the pleura. (**B)** Time to recurrence depending on the distance to adjacent vessels. (**C)** Local recurrence regarding the possible influence of the distance to the bronchus.

Ten pulmonary lesions were adjacent to the pleura (≤ 1 cm), while 119 showed a distance ≥ 1 cm. 2 of the 10 pleural lesions showed a local recurrence. 17 of 119 lesions with a distance ≥ 1 cm showed a local recurrence (Figure 3A, Table 1). There was no significant influence regarding the distance of pleura to the local recurrence (*p* = 0.807, log-rank).The distance from vessels and bronchi showed no statistically significant effect on local recurrence rate (*p* = 0.812, log-rank; *p* = 0.820, log-rank) (Figure 3B, 3C; Table 1).

The median measurable diameter of a vessel located near a treated lesion was 0.4 cm (mean, 0.6, 0.1–3 cm) and the median measurable diameter of an adjacent bronchus was 0.4 cm (mean, 0.5, 0.2–2 cm). These possible relevant parameters showed no significant influence.

**Figure 4:**
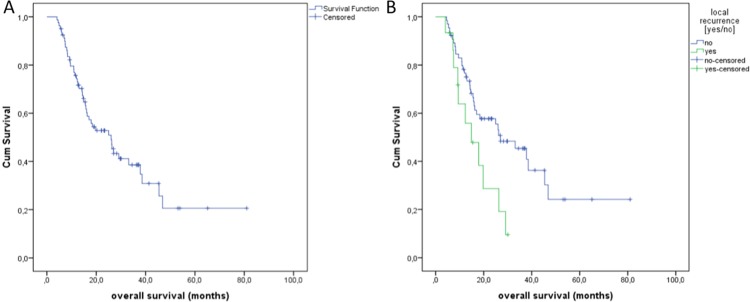
(**A)** Overall Survival (OS) of all treated primary and secondary pulmonary lesions with a median of 27 months. (**B)** OS depending on the local recurrence.

**Figure 5:**
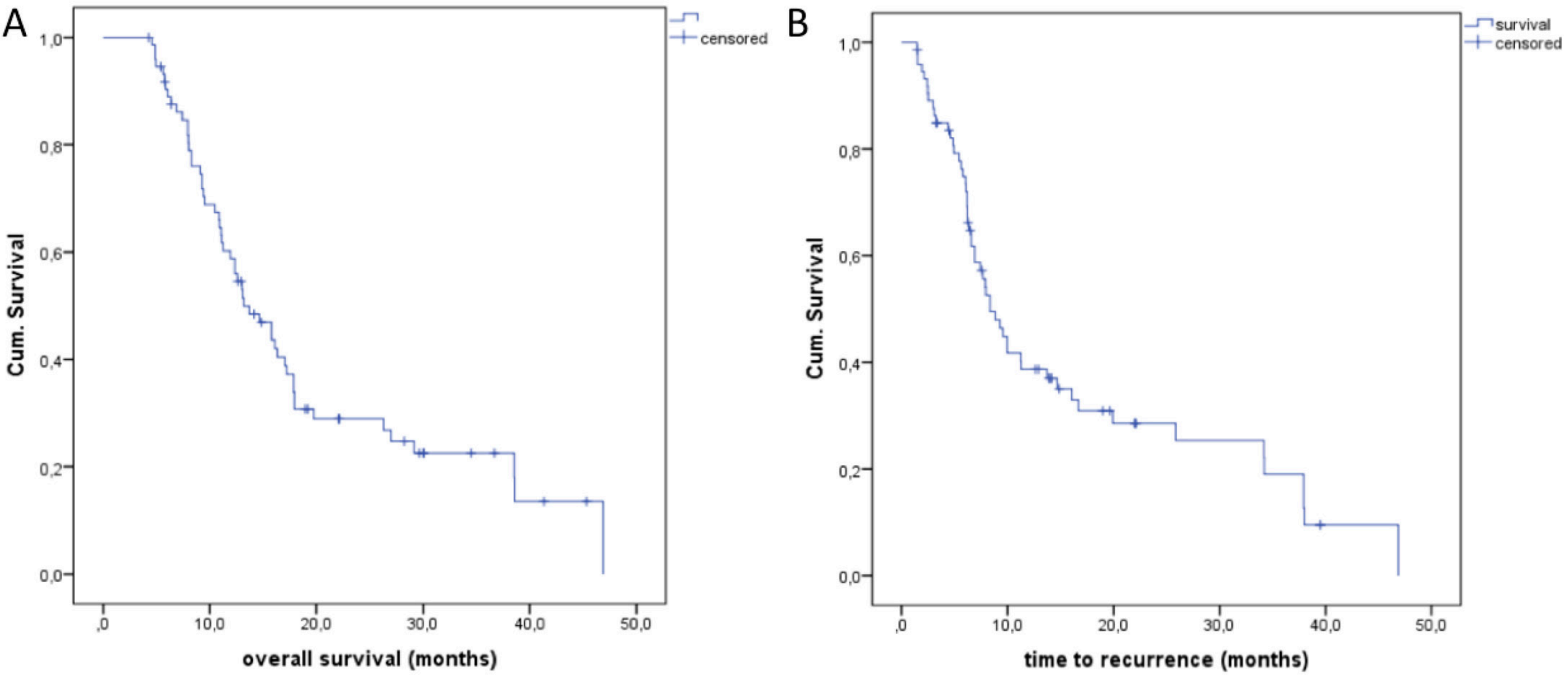
(**A**) Overall Survival (OS) of the colorectal lung metastases with a median of 13 months. (**B)** Time to recurrence (TTR) of lung metastases of colorectal cancer with a median of 8 months.

The median OS after treatment was obtained after a period of 27 months (Figure 4A). The median OS in the group of local recurrence was only 14.9 months (Figure 4B). Local recurrence showed a significant influence regarding the overall survival (*p* = 0,020, log-rank). In a subgroup analysis, we evaluated the OS of the patients with 74 treated colorectal pulmonary metastases. In these cases, the median OS was 13 months (Figure 5A) and the median time to recurrence was 8 months (Figure 5B).

### Safety and adverse events

Overall, pneumothorax in 19 cases (14.7%) and pleural effusion in 2 patients (1.6%) were the leading complications in this series. Minor complications were recorded after 18 procedures (14%) (Tables 2, 3). There were 5 major complications (3.9%) in conjunction with pneumothorax and pleural effusion that required treatment via placement of a small-bore catheter in one patient (0.8%) and a larger chest tube placement in 2 patients (1.6%), rated as CTCAE Grade II and III (Tables 3, 4). One patient suffered from a painful skin burn (0.8%) as a major complication from cutaneous adhesive electrode grounding during the ablation session, rated as CTCAE Grade III. A postprocedural infection caused a pulmonary abscess cavity in one patient (0.8%) requiring systemic antibiotic therapy, rated as CTCAE Grade II. Our study showed no Grade 4 and 5 CTCAE due to the ablation.

**Table 2:** Adverse events after the treatment of primary and secondary lung malignancies with CT-guided radiofrequency ablation

Number of adverse events (*n*)	23
pneumothorax (n; %)	19; 147%
pleural effusion (n; %)	2; 1.6%
skin burning (n; %)	1; 0.8%
infection/abscess (n; %)	1; 0.8%
**Number of *major* events (n, %)**	**5; 3.9%**
**Number of *minor* events (n, %)**	**18; 14.0%**

**Table 3:** Moderate and severe adverse events (Grade II and III) after RF ablation of pulmonary lesions rated with CTCAE (Common terminology criteria for adverse events)

CTCAE Grade:	II	III	IV	V
**Pneumothorax**	1	0	0	0
**Pleural effusion**	0	2	0	0
**Skin Burning**	0	1	0	0
**Pulmonary infection**	1	0	0	0

**Table 4:** Clinical characteristics of patients with primary and secondary pulmonary lesion

Total number of patients (*n*)	79
**Sex (m; w)**	m = 47; w = 32
**Median age (y), range**	(65.1; 36–83)
**Total pulmonary lesions (**n)	**129**
**Histology of Primary tumor**	
Colorectal cancer (n)	74
Malignant melanoma (n)	13
Renal cell carcinoma (n)	13
Non-small-cell lung cancer (n)	5
Other primary malignancies (n)	24
**Median size** of pulmonary lesions, range (cm)	1.2 cm, (0.5–3.0 cm)
**Previous lung surgery (*n*)**	18; 22, 5%
**Median treatment time (min)**	17 (10–30)

## DISCUSSION

Different local therapies are available for patients with NSCLC and secondary oligometastatic disease, including stereotactic body radiation therapy (SBRT, 97.6% LTC rate) [10], stereotactic radiosurgery with CyberKnife (95% LTC rate) [11] as well as image-guided thermal and non-thermal tumor ablation [12, 13]. Numerous studies in the literature demonstrate the outcome of RFA in pulmonary lesions. A most recently published study by Nour-Eldin et al. compared LITT, RFA and MWA in the treatment of non-colorectal lung metastases. The study reported a LTC rate of 70.6% for LITT, 79.3% for RFA and 90.5% for MWA. Pneumothorax was detected in 22.73% for LITT, 14,23% for RFA and 22.16% for MWA [14]. Regarding those satisfying results, RFA still has its impact as an ablative oncologic therapy method.

The presented study, with a LTC of 85.3% and a complication rate of 14% minor complications and 4% major complications, could contribute and widely confirm the data in the literature. Yamakado et al. achieved a LTC of 83% in 155 treated lung metastases from colorectal cancer in a Japanese multicenter study [15]. A study on 153 patients by Simon et al. differentiated between large and small lesions (< 3 cm) showing an important positive influence on survival curves in conjunction with smaller lesions [6]. Those results are consistent with our study, demonstrating a significant influence of tumor size on local tumor progression. De Beare et al. reported the largest series today of RFAs in 1037 lung metastases from a variety of primary sites with a median OS of 62 months. The location of primary disease, disease-free interval (DFI), size > 2 cm, and number of metastases were significantly associated with OS in uni- and multivariate regression analyses [16].

For lung metastases, OS rates after RFA are within the range of best results obtained after surgical resection, with 5-year OS rates of 53.5% for Iida et al. [17] in a multicenter registry, in between 27% and 68% in a meta-analysis by Gonzalez et al. [18]. The median OS of our population was 27 months. Regarding the tumor size, OS was quite similar in the patient group 0.5–1 cm (26.2 months) and 1–2 cm (26.0 months). Patients with a treated size of 2–3 cm showed a median overall survival of 16.3 months. The subgroup of patients with colorectal cancer had a median OS of 13 months. The better outcomes obtained by de Baere et al. are explained by the restricted inclusion criteria, resulting in more favorable predictive factors.

Various research have demonstrated efficient LTC regarding small tumor size, but there are only few studies that evaluate the influence of adjacent vessels and bronchi, the distance to the pleura and the diameter of nearby vessels and bronchi, as analyzed in the presented study.

Contrary to other studies, neither contact to vessels and bronchial tubes, nor vessel and bronchi diameter showed any influence on treatment success in our study. For example, Schneider et al. [19] detected lung neoplasms in a central or hilar location most frequently occurring local ‘heat sink effect’, and viable tumor cells next to peripheral vessels remaining after ablation procedures by histologic proof.

Histological type as another important factor Hiraki et al. [20] have been considered without statistical validation. A univariate analysis by Garetto et al. [7] documented lower LTC for the treatment of NSCLC compared to pulmonary metastases. In view of the fact that our study counted just 5 patients with NSCLC, our series was inappropriate to undergo statistically significant subgroup analysis, which has to be noted as a limitation.

In our study, we recorded minor complications in 14% and major complications in 4%, without worsening of pulmonary function or CTCAE Grade IV or V complications. Other series stated a similar safety profile with pneumothorax and pleural effusion as the most frequent complications [5, 7]. Periprocedural mortality rate was less than 1% [21] and periprocedural morbidity varied between 15.5% and 55.6% in the literature [22, 23]. Major complications ranged from 8% to 12% [22, 23] in former clinical trials. In the majority of cases, complications after RFA were minor. Nevertheless, rare and serious complications can occur, so radiologists should be familiar with different types of complications. Quick management is essential for a successful treatment. In individual cases, infection or hemorrhage occurred from cavity formation due to tumor colliquation or postinterventional pneumonia stated in up to 30% of cases [24]. Skin burns around the grounding pads as reported in one patient from our series was described in correlation with monopolar radiofrequency ablation. Thus, temperature monitoring at the grounding pads during the procedure is necessary [25].

A prospective multicenter trial from Japan in 2016 analyzed the incidence and grade of AEs using the CTCAE in 33 patients. Two patients showed pleural effusion, which was rated as CTCAE Grade III. One patient had a transient hypoxia with SaO_2_ < 88%. Pneumothorax occurred in 12 of 33 patients (36%). Two of them needed a chest tube placement (6.7%). Gobara et al. hypothesized that the observed incidence was due to a high proportion of the patient population having a previous history of thoracic surgery. They claimed that the absence of prior surgical operations might be a risk factor for chest tube placement for pneumothorax [26].

Our study had some limitations. This retrospective analysis was based on a heterogeneous group comprising both primary and secondary lung neoplasms and different former and adjunctive therapies such as chemo- and radiotherapy with unknown influence on the treatment success. Moreover, follow-up visits only consisted of image-guided assessment associated with lesion size, lesion geometry and lesion enhancement, without consideration of histologic proof for treatment completeness. CT scans were related with different patient positioning in the image plane for axial imaging. Other studies evaluated the outcome by using FDG-PET/CT to assess treatment outcomes [27, 28]. With regards to overall survival endpoint, life quality assessment would have been interesting to complete the evaluation of achievements.

Despite these limitations, this study was able to confirm technical success, efficacy and safety of RFA in the treatment of pulmonary malignancies according to other clinical trials in recent years. Furthermore, a feature of this study was the evaluation of different predictive factors, influencing the clinical outcome. In contrast to the distance to pleura, vessel or bronchus, and the vessel and bronchus diameter, the tumor size had a significant influence. This finding underlines the necessity of further technical development of RF technology in the future to improve this already well established and well tolerated method.

The results of the presented study contribute to the statement that in the future lung surgery for small-size oligometastatic lung disease will be replaced by low-invasive techniques in selected patients [29]. Future treatment strategies should combine surgical, systemic and interventional options for individual patient-tailored success.

## MATERIALS AND METHODS

### Patient characteristics and study design

This retrospective single-center study is based on a sample of 79 patients with 129 pulmonary tumor lesions treated within a period of 6 years at a German university clinic (Table 4). Included in the study were 47 men (60%) and 32 women (40%) with a median age of 65.1 years (range 36–83 years). All patients exhibited primary and secondary lung malignancies. In detail, we treated 74 metastases of colorectal cancer, 13 malignant melanoma lesions, 13 renal cancer lesions, 5 primary lung malignancies and 24 tumors of different entities. Pulmonary target lesions ranged from 0.5 to 3.0 cm (median 1.2 cm).

Indication was determined on the basis of multidisciplinary assessment with participation by an interventional radiologist, oncologist, surgeon and pathologist. Inclusion criteria for the ablation were: (a) patients with inoperable pulmonary lesions, (b) poor candidates for surgery due to medical conditions or unsuitable candidates due to advanced cancer related morbidities, (c) patients considered unfit for radiotherapy or chemotherapy, (d) lesions with a maximal axial diameter < 4 cm, (e) pulmonary tumor lesions infiltrating the chest or mediastinal structures and (f) Eastern Cooperative Oncology Group (ECOG) performance status 0, 1 or 2 and platelet counts > than 100 × 10^9^/L.

Exclusion criteria were: (a) patients considered high-risk for RFA due to major comorbidities, (b) more than three lesions per lung, (c) ECOG performance status of more than 2 and (d) platelet counts less than or equal to 100 × 10^9^/L. Clinical history that revealed former thoracic surgical interventions was not an exclusion criterion and was shown in 18 patients (22.5%).

All patients provided written informed consent of the ablation therapy and its possible complications. We had an IRB approval for the retrospective study design.

### Interventional procedure

Percutaneous radiofrequency ablation (PRFA) was performed under image guidance by using a 16- and 64-row CT scanner (Toshiba Aquilon 16, Toshiba Prime 64, Japan). Ablation energy was produced by an RF-generator (RF3000^®^, Boston Scientific, USA) with a maximum power of 200 W and transmitted by impedance-based expandable needle electrodes (LeVeen^®^, Boston Scientific, Nattick, MA). All patients underwent intravenous sedation with Midazolam and Fentanyl on demand and local anesthesia with Xylocain.

Under CT guidance, a suitable positioning of the patient according to an optimal skin entry site was chosen, allowing the shortest and safest path to reach the target lesion without affecting endangered anatomic structures such as pleura, blood vessels or bronchial tubes. The needle electrode was inserted under conditions of cutaneous sterility. After correct placement of the needle electrode in the tumor, the RF-ablation procedure was started under step-wise settings following different algorithms depending on the localization of the tumor and according to pleural distance. After roll-off in impedance-control mode, ablation was performed in a second cycle with energy lowered to a 70%-level compared to former maximal power output. The defined ablation area always encompassed a standard 0.5 cm safety margin to avoid residual local tumor progression. Following the thermal ablation procedure, the needle electrode was pulled back under track ablation with an energy of 10 W to destroy possible transmitted tumor cells along the pass channel and to prevent pleural bleeding complications.

To conclude the procedure, a CT scan of the ablation area was performed upon request to assure technical success and to rule out early complications. After treatment, patients were kept under clinical observation to monitor their general and cardiorespiratory condition. To follow the possible evolution of complications such as pneumothorax, chest X-rays were obtained at 4 hours and 24 hours after intervention in all cases.

### Assessment of outcome variables

The primary endpoint of our study was local tumor control (LTC). Secondary endpoints were technical success and safety as well as overall survival (OS). Technical success here refers to the correct placement of the needle electrode and procedural settings fulfilling the standard ablation protocol.

**Figure 6:**
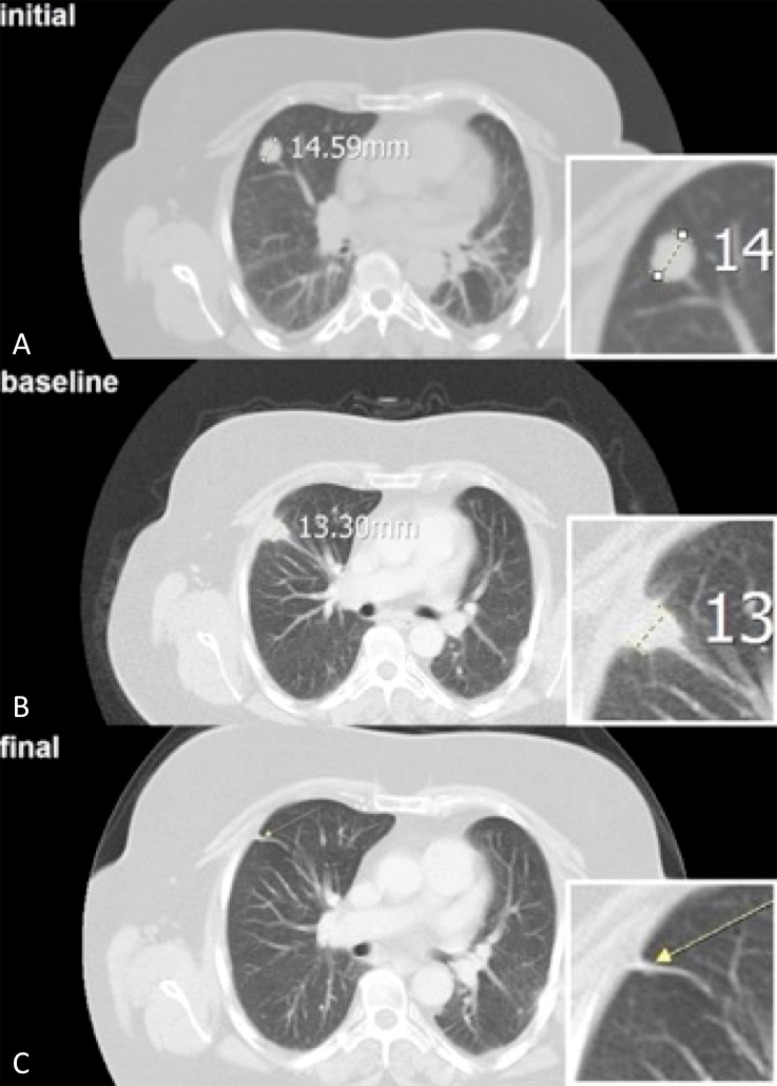
(**A)** The axial initial CT scan of the right lung shows a 1.5 cm large tumor of a patient with a colorectal cancer metastasis. (**B)** After the CT-guided radiofrequency ablation (RFA) of the pulmonary lesion, the baseline CT scan shows a typical surrounding pleural reaction. (**C**) Complete remission (CR) after a follow-up period of 25 months.

Follow-up visits coincided with a baseline CT scan within 1 month after RFA treatment, a CT scan every three months following and a final CT scan as control (Figure 6A–6C). We included patients with a minimum of two follow-up CT scans. Because the RFA causes a coagulation necrosis in the ablation zone presented as a high density area in CT, the follow-up CT scans were compared with the baseline scan shortly taken after the ablation to exclude a false positive tumor progression. Treatment evaluation, i.e. changes of the high density surrounding area or tumor sizes were analyzed by using the Response Evaluation Criteria in Solid Tumors (RECIST 1.1) [30], according to the follow-up scheme of the RAPTURE study [5]. Two independent radiologists with 7 and 13 years’ experience in oncological/interventional radiology evaluated the CT scans. In cases of discordance, results were obtained by consensus. Based on CT analysis, LTC was evaluated by axial lesion size measurement in longest diameter, lesion geometry and lesion enhancement using *Infinitt PACS^®^* as the established software for radiological analysis and picture diagnostics. Target tumors, showing at least a 30% decrease in longest diameter compared with the diameter measured at baseline CT, no evidence of tumor growth and no evidence of contrast enhancement, were assumed to have undergone partial and complete remission (PR, CR). Target tumors seen in follow-ups that showed evidence of an increase in longest diameter of at least a 20% tumor growth, or intratumoral contrast uptake, were assumed to have a progressive disease (PD) as a consequence of an incomplete ablation.

Potential complications during or after the procedure were monitored and reported. Safety assessment dealing with procedure-related complications were divided into *major* and *minor* complications. Major complications were pneumothorax and pleural effusion that required chest tube placement; in general terms, major complications limited the patient’s general condition and especially restricted lung function to a certain extent, making repeated treatment necessary. Minor complications appeared less dangerous, self-limiting and tolerable, so the patient only required observation without the need of intervention. We analyzed the adverse events (AEs) using the “Common Terminology Criteria for Adverse Events Version 3.0” (CTCAE 3.0).

### Assessment of predictive factors

**Figure 7:**
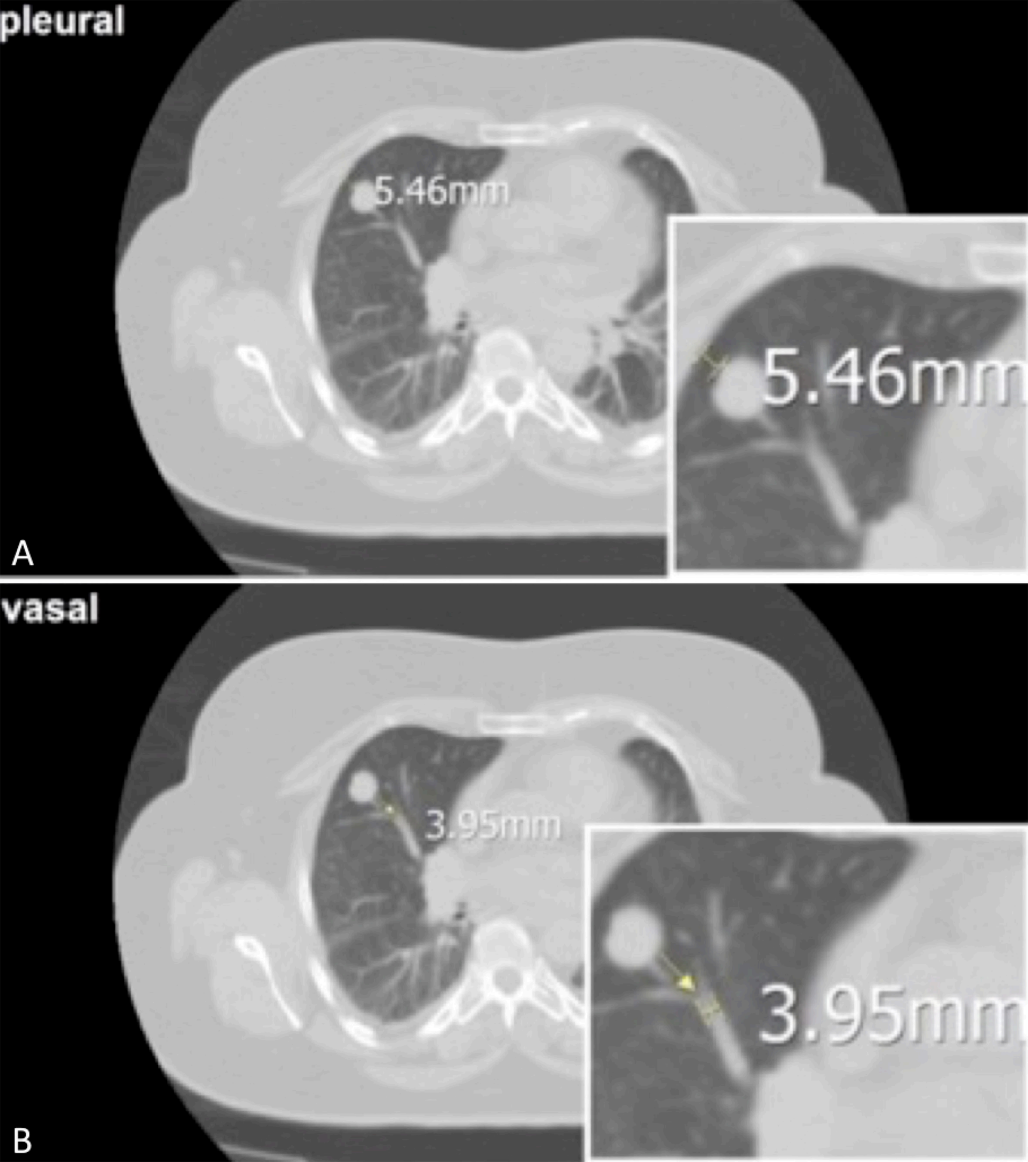
(**A)** The axial CT scan of a pulmonary lesion shows the distance to the pleura (5 mm) as a possible influence. (**B)** and the distance to an adjacent vessel (4 mm).

In order to identify possible predictive factors co-variating with the success of treatment, we examined the influence of a) initial lesion size, b) distance to vessels, c) bronchi and d) pleura (Figure 7A). We defined the distance of a pulmonary lesions to the next measurable vessel (Figure 7B) or bronchus (0–0.5 cm; 0.5–2 cm; > 2 cm). We also evaluated e) the vessel and f) bronchial caliber both contributing to the local ‘heat sink effect’.

### Statistical methods

Our data was collected from our internally established data base ASENA^®^ (LoeScap Technology GmbH) and tabulated in a Microsoft Excel^®^ 2007 (MS Windows^®^) worksheet. Statistical analysis was performed using SPSS^®^ 21 (MS Windows^®^). The Kaplan-Meier method was used to estimate survival functions. Median survival estimates were reported with 95% confidence intervals. Comparisons of survival functions were performed by using both the log-rank test and the Breslow test. Correlations between the variables were comprehensively analyzed by means of Cox regressions. *P* < 0.05 was considered to indicate statistically significant difference.
